# Comparison of human and Drosophila atlastin GTPases

**DOI:** 10.1007/s13238-014-0118-0

**Published:** 2014-11-20

**Authors:** Fuyun Wu, Xiaoyu Hu, Xin Bian, Xinqi Liu, Junjie Hu

**Affiliations:** 1 Department of Genetics and Cell Biology, College of Life Sciences, Nankai University, Tianjin, 300071 China; 2 Tianjin Key Laboratory of Protein Sciences, Tianjin, 300071 China; 3 Department of Biochemistry and Molecular Biology, College of Life Sciences, Nankai University, Tianjin, 300071 China; 4 State Key Laboratory of Medicinal Chemical Biology, Tianjin, 300071 China; 5 National Laboratory of Biomacromolecules, Institute of Biophysics, Chinese Academy of Sciences, Beijing, 100101 China

**Keywords:** endoplasmic reticulum, membrane fusion, atlastin, GTPase, X-ray crystallography

## Abstract

Formation of the endoplasmic reticulum (ER) network requires homotypic membrane fusion, which involves a class of atlastin (ATL) GTPases. Purified Drosophila ATL is capable of mediating vesicle fusion *in vitro*, but such activity has not been reported for any other ATLs. Here, we determined the preliminary crystal structure of the cytosolic segment of Drosophila ATL in a GDP-bound state. The structure reveals a GTPase domain dimer with the subsequent three-helix bundles associating with their own GTPase domains and pointing in opposite directions. This conformation is similar to that of human ATL1, to which GDP and high concentrations of inorganic phosphate, but not GDP only, were included. Drosophila ATL restored ER morphology defects in mammalian cells lacking ATLs, and measurements of nucleotide-dependent dimerization and GTPase activity were comparable for Drosophila ATL and human ATL1. However, purified and reconstituted human ATL1 exhibited no *in vitro* fusion activity. When the cytosolic segment of human ATL1 was connected to the transmembrane (TM) region and C-terminal tail (CT) of Drosophila ATL, the chimera still exhibited no fusion activity, though its GTPase activity was normal. These results suggest that GDP-bound ATLs may adopt multiple conformations and the *in vitro* fusion activity of ATL cannot be achieved by a simple collection of functional domains.

## INTRODUCTION

In eukaryotic cells, the endoplasmic reticulum (ER) is composed of interconnected tubules and sheets (Shibata et al., 2006). The merging of ER membranes, i.e. homotypic fusion, is required for ER network formation and mediated by a dynamin-like membrane bound GTPase family known as atlastins (ATLs) (Hu et al., 2009; Orso et al., 2009). No ATL has been found in yeast and plant cells, but a similar class of GTPases, named Sey1p or RHD3, plays an analogous role in ER morphogenesis (Hu et al., 2009; Anwar et al., 2012; Zhang et al., 2013). Depletion of Drosophila or zebrafish ATL results in neuronal defects (Lee et al., 2008; Lee et al., 2009; Fassier et al., 2010). Deletion or mutation of RHD3 leads to defects in plant growth and short and wavy root hairs (Schiefelbein and Somerville, 1990; Wang et al., 1997; Stefano et al., 2012). Depletion or mutation of human ATL1, a dominant form in the central nervous system, causes unbranched ER at the cellular level (Hu et al., 2009) and hereditary spastic paraplegia (HSP), a neurodegenerative disease characterized by axon shortening in corticospinal motor neurons and progressive spasticity and weakness of the lower limbs, at the organism level (Zhao et al., 2001; Salinas et al., 2008).

Several assays have been developed to study the fusogenic activity of these GTPases. First, yeast cells lacking Sey1p and either Yop1p or Rtn1p, members of the ER tubule-forming protein family, exhibit abnormal ER morphology (Hu et al., 2009). Restoration of the ER network can then be monitored upon expression of any ER fusogen candidates (Anwar et al., 2012; Zhang et al., 2013). Second, ER fusion can be followed during mating of haploid yeast cells carrying cytosolic and ER markers (Anwar et al., 2012). Deletion of Sey1p significantly reduces the fusion process, whereas expression of a functional ER fusogen, such as human ATL1 or RHD3 (Anwar et al., 2012; Zhang et al., 2013), can rescue this defect. Moreover, purified drosophila ATL has been reconstituted into proteoliposomes, and signals of lipid mixing in the presence of magnesium and GTP provide direct evidence for membrane fusion mediated by ATL (Orso et al., 2009; Bian et al., 2011). Using this assay, Sey1p and members of the RHD3 family have been confirmed to mediate fusion *in vitro*. However, drosophila ATL is the only ATL that has been reported to have fusogenic activity *in vitro*.

**Figure 1:**
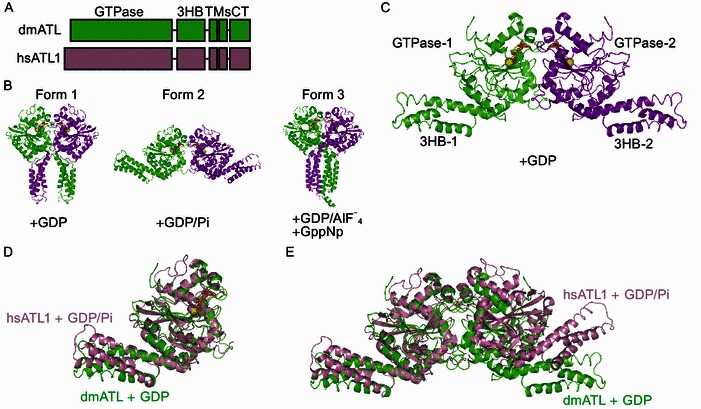
Crystal structure of the cytosolic domain of Drosophila ATL. (A) Schematic diagrams of Drosophila ATL and human ATL1. (B) Human ATL1 dimers observed in crystal form 1 (PDB code: 3QNU, left, GDP-bound), form 2 (PDB code: 3QOF, middle, GDP-bound in the presence of inorganic phosphate), and form 3 (PDB code: 4IDO, right, GDP/AlF_4_-bound or GppNp-bound) are shown. The protomers in the dimer are shown in green and purple cartoon representation. (C) Structure of the GDP-bound form of Drosophila ATL. The protomers in the dimer are shown in green and purple cartoon representation. GDP is shown in orange stick representation, and magnesium ion is shown as a yellow sphere. 3HB, three-helix bundle. (D) Structural comparison of human and Drosophila ATLs. Human ATL1 (hsATL1) is shown in pink and Drosophila ATL (dmATL) is shown in green. GDP in dmATL is shown in orange, and magnesium ion is shown in yellow. (E) As in C, but with a dimer. Superposition was performed using the protomers on the left

ATL contains an N-terminal GTPase and a three-helix bundle (3HB), followed by two closely spaced transmembrane (TM) segments and a C-terminal tail (CT) (Fig. 1A). In an effort to understand the ATL-associated fusion mechanisms, three crystal structures of the N-terminal cytosolic domain of human ATL1 have been determined (Bian et al., 2011; Byrnes and Sondermann, 2011). In all three forms of ATL, the molecule forms a dimer with the GTPase domains facing each other, but the position of the 3HB differs (Fig. 1B). In form 1, ATL1 is in complex with GDP, the two 3HBs associate with the paired GTPase domains and form a crossover conformation. In this case, the connecting TMs would have to sit in the same membranes; thus, the structure corresponds to a “post-fusion” state in which the membranes have already fused. In form 2, ATL1 is also GDP-bound, but in the presence of high concentration of inorganic phosphate. The two 3HBs associate with their own GTPase domains and point in opposite directions. This structure implies that two ATL molecules likely sit in apposing membranes, corresponding to a membrane-tethered “pre-fusion” state. In form 3, ATL is crystallized with either GDP/AlF_4_ or GppNp. The two 3HBs come even closer than in form 1. Collectively, ATL-mediated fusion requires dimerization resulting from GTP binding and conformational changes induced by GTP hydrolysis (Bian et al., 2011; Byrnes and Sondermann, 2011; Hu et al., 2011; Lin et al., 2012)

In addition to the N-terminal cytosolic domain, the TM and CT have also been shown to play important roles in fusion (Liu et al., 2012). The TM of ATL appears to be more than a membrane anchor, as deletion or replacement of the TM region results in a loss of fusion activity. The CT of ATL forms an amphipathic helix that binds and destabilizes the membranes to facilitate fusion. A synthetic peptide corresponding to parts of the CT of Drosophila ATL was able to restore fusion defects in the tailless ATL mutant. The importance of ATL-CT is consistent with the fact that a lack of CT in human ATL1 mutants causes HSP (Liu et al., 2012).

Drosophila ATL has been used extensively to study membrane fusion *in vitro*, but structural information about ATL has been obtained purely from human ATL1. Whether human ATL can mediate fusion *in vitro* is also not clear. Here, we determined the structure of the N-terminal cytosolic domain of Drosophila ATL and compared fusogenic features of human and Drosophila ATL.

## RESULTS

### Crystal structure of the cytosolic domain of Drosophila ATL

To gain insight into Drosophila ATL-mediated membrane fusion, we determined the crystal structure of cytATL. Residues 1**–**422 were expressed in *E. coli*, purified, and crystallized in the presence of GDP. A centered monoclinic crystal form was used to determine the structure at 2.87-Å resolution by molecular replacement using the human ATL1 structure (PDB code: 3QOF) as a search model (Table 1). Despite numerous trials for crystal optimization, we were unable to improve the data quality; the final resolution is acceptable for phase determination and preliminary structural interpretation. One ATL molecule was present in the asymmetric unit, but a crystallographic dimer was observed along one of the two-fold symmetry axes.

**Table 1:** Data collection and refinement statistics

**Data collection**	
Space group	*C121*
Wavelength (Å)	1.54
Unit cell	
a, b, c (Å)	71.62, 63.91, 107.76
α, β, γ (°)	90, 101.6, 90
Molecules/ASU	1
Resolution range (Å)^a^	50–2.87 (2.92–2.87)
Completeness (%)^a^	91.93 (80.49)
Redundancy^a^	3.1 (2.5)
No. of total reflections	31354
No. of unique reflections	10190
*I*/*σ*^a^	26.4 (3.8)
* R*sym (%)^a,b^	8.1 (31.5)
**Refinement statistics**	
Resolution (Å)	2.87
No. of reflections	10185
*R*_work_/*R*_free_ (%)^c^	27.30/36.40
No. of atoms	
Protein	2928
Ligands	29
Water	6
B-factors (Å^2^)	
Protein	41.49
Water	41.64
Ligands	28.41
r.m.s.d.	
Bond length (Å)	0.009
Bond angle	1.487
**Ramachandran analysis**	
Most favored (%)	65.0
Additional allowed (%)	27.9
Generously allowed (%)	4.7
Disallowed (%)	2.4

^a^ Values in parentheses are for the highest resolution shell

^b^*R*sym = Σ|I − <I>|/Σ<I>, where I is the observed intensity, and <I> is the average intensity of multiple symmetry-related reflection observations

^c^*R* = Σ_hkl_|*F*_obs_| − |*F*_calc_|/Σ_hkl_|*F*_obs_|. *R*_free_ was calculated from 5% of the reflections excluded from refinement

Similar to human ATL1, the structure of Drosophila cytATL revealed a GTPase domain and a 3HB connected by a linker region (Fig. 1C). In the GDP-bound dimer, the GTPase domains interact with each other. The two 3HBs dock to their own GTPase domains and point in opposite directions. This configuration is very similar to form 2, or the “pre-fusion” state, of human ATL1 (Fig. 1B). In fact, the cytATLs of human and Drosophila are virtually superimposable (Fig. 1D). Only part of the 3HB showed appreciable differences between the two structures (Fig. 1D). When the dimers of human and Drosophila ATL were compared, the relative position between two protomers was slightly different (Fig. 1E). Notably, the dimer of Drosophila ATL was observed with GDP, but the similar dimer of human ATL1 was seen with GDP and a high concentration of inorganic phosphate. When only GDP was added, a completely different human ATL1 dimer (form 1 in Fig. 1B) was formed. In addition, when average B-factors were calculated for the GTPase domain and the 3HB in our structure (37.84 for GTPase and 60.59 for 3HB), the results suggested that the 3HB exhibited significantly higher flexibility. These results suggest that the 3HB of ATL may adopt more than one conformation upon GDP binding.

**Figure 2:**
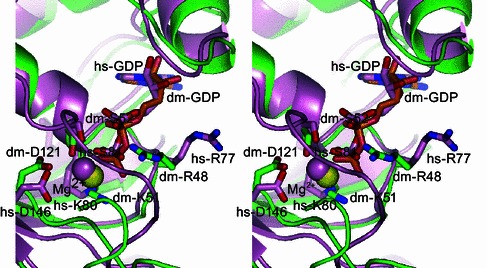
Comparison of the active site. Stereoview of the superimposed active sites of Drosophila ATL (colored as in Fig. 1C) and human ATL1 (in pink, PDB ID code 3QOF). Critical residues are highlighted. The nucleotides are shown in stick representation and the magnesium ions as spheres

The active site of Drosophila ATL is mostly very similar to that of human ATL1 (Fig. 2), where conserved residues from four signature motifs engage the nucleotide in the presence of magnesium ion. Notably, R48 of Drosophila ATL points towards GDP, while the equivalent Arginine in human ATL1 (R77) points away from GDP, and forms a salt bridge with a Glutamic acid in the pairing GTPase (Bian et al., 2011). In the recent human ATL1 structures, where GppNp or GDP/AlF_4_ were added, R77 points at the nucleotides (Byrnes et al., 2013). Thus, this Arginine was proposed to flip between these conformations during GTP hydrolysis: it might facilitate GTPase dimer formation in the outward position and act as an Arg-finger in the inward position. Even though the electron density for R48 is poor in our structure, it is consistent that this critical arginine has the flexibility to switch between conformations, especially in the GDP-bound state.

### Drosophila ATL can functionally replace human ATLs

**Figure 3:**
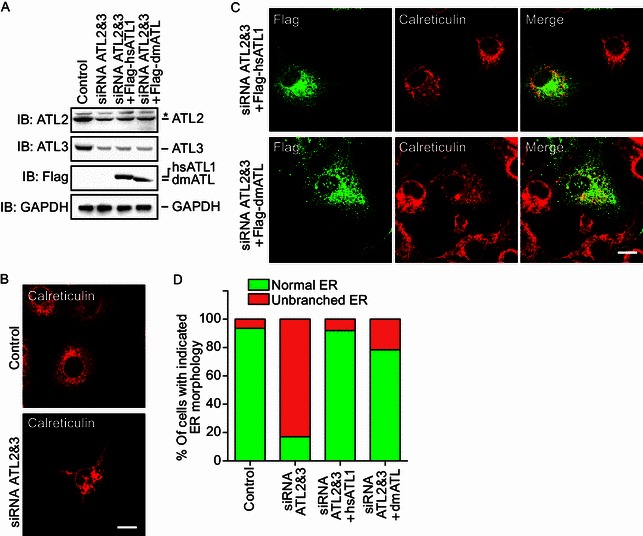
Functional tests of Drosophila ATL in mammalian cells. (A) COS-7 cells were transfected with siRNA oligonucleotides as indicated. Levels of endogenous ATL2 and ATL3 or exogenously expressed Drosophila ATL and human ATL1 were determined by immunoblotting. GAPDH was used as a loading control. Asterisk (*) indicates a non-specific band. (B) The ER morphology of COS-7 cells was visualized using calreticulin, an endogenous luminal ER protein, and indirect immunofluorescence using a confocal microscope. Scale bar = 10 μm. (C) As in (B), but with double-depleted COS-7 cells transfected with Flag-human ATL1, or Drosophila ATL. (D) The ER morphology of samples shown in (B) and (C) was categorized as “normal” or “unbranched”. A total of 80–150 cells were counted for each sample. All graphs are representative of three repetitions

To analyze the function of Drosophila ATL, we tested whether it can replace ATLs in mammalian cells. We first used small interference RNA (siRNA) to deplete major ATL proteins in COS-7 cells and visualized the ER morphology using calreticulin as a marker. The COS-7 cell line expresses little ATL1 but sufficient ATL2 and ATL3. Consistent with previous results, when ATL2 and ATL3 were partially knocked down in COS-7 cells, the ER in most of the cells exhibited unbranched morphology (Fig. 3A, 3B, and 3D), indicating defects in ER fusion. As expected, expression of human ATL1 efficiently restored the ER network in double-depleted cells (Fig. 3A, 3C, and 3D). When Drosophila ATL was expressed at a comparable level, significantly less cells exhibited unbranched ER phenotype (Fig. 3A, 3C, and 3D). These results indicated that Drosophila ATL and human ATL1 are equally active in maintaining the ER network.

### Dimer formation and GTP hydrolysis of Drosophila and human ATLs

**Figure 4:**
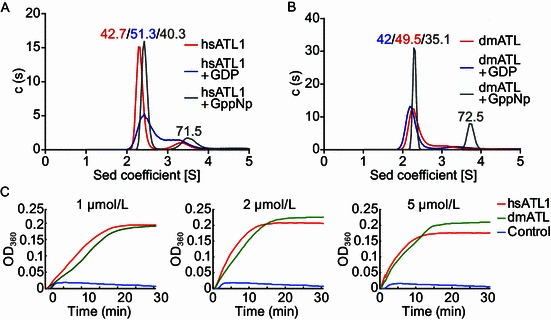
Biochemical comparison of cytATLs. (A) Nucleotide-dependent dimerization of the N-terminal cytosolic domain of hsATL1 was determined at a concentration of 0.03 mmol/L by analytical ultracentrifugation in the presence of the indicated nucleotides. (B) As in (A), but with dmATL. (C) The GTPase activities of 1 μmol/L, 2 μmol/L, and 5 μmol/L of N-terminal cytosolic domains of human ATL1 (hsATL1) and Drosophila ATL (dmATL) were measured by phosphate release at saturating concentrations of GTP (0.5 mmol/L). All graphs are representative of three repetitions

ATL-mediated fusion has been shown to require GTP-dependent dimerization between ATL molecules in apposing membranes, and GTP hydrolysis drives conformational changes in ATL. To test whether Drosophila and human ATLs differ in dimerization tendencies upon GTP binding, we compared the dimerization state of purified cytATLs using analytic ultracentrifugation (AUC). As shown previously (Bian et al., 2011), but to a lesser extent, human cytATL1 formed some dimer in the presence of GppNp, a non-hydrolyzable analog of GTP, at a concentration of 0.03 mmol/L (Fig. 4A). A similar tendency of dimerization was observed when Drosophila cytATL was tested in the presence of GppNp (Fig. 4B). In contrast, little dimer was seen when both proteins were tested at the same concentrations but with GDP (Fig. 4A and 4B). We then compared the GTPase activities of purified cytATLs. When the GTPase assay was performed using Drosophila or human cytATLs at a concentration of 1 μmol/L, similar rates of GTP hydrolysis were detected (Fig. 4C, left panel). The same increase in hydrolysis rates was measured when higher concentrations (2 μmol/L and 5 μmol/L) of the proteins were tested (Fig. 4C, middle and right panels). Taken together, these results suggest that Drosophila and human ATLs display the same tendencies in nucleotide-dependent dimerization and the same GTP hydrolysis ability.

### Testing human and Drosophila ATLs in a fusion assay

**Figure 5:**
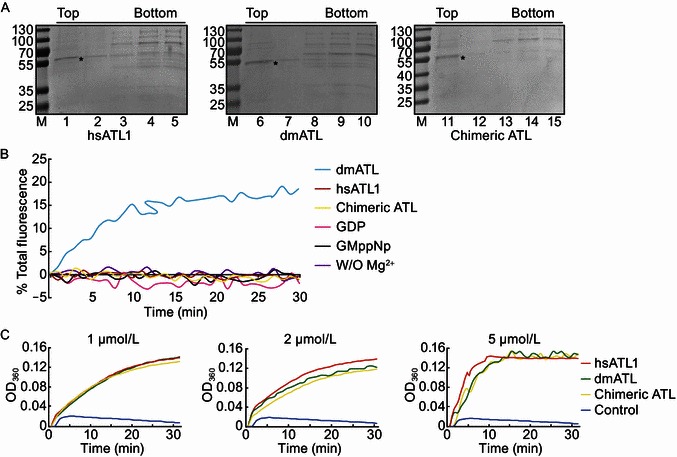
Comparison of membrane fusion *in vitro*. (A) Full-length ATLs were reconstituted into proteoliposomes. Flotation in a sucrose gradient indicated efficient reconstitution of the proteins. (B) Full-length ATLs were reconstituted at equal concentrations into donor and acceptor vesicles. The lipid to protein ratio used for each protein is indicated. Fusion was monitored by dequenching the NBD-labeled lipids present in the donor vesicles and was initiated by the addition of GTP. Control experiments were performed using chimeric ATL in the absence of Mg^2+^ or the presence of GDP or GppNp instead of GTP. (C) The GTPase activities of purified full-length ATLs (1 μmol/L, 2 μmol/L, and 5 μmol/L) were measured by phosphate release at saturating concentrations of GTP (0.5 mmol/L). All graphs are representative of three repetitions

To compare the membrane fusion activity of full-length ATLs, we used a previously established *in vitro* lipid-mixing assay. Drosophila and human ATLs were purified and reconstituted at equal concentrations into donor and acceptor proteoliposomes (Fig. 5A). The donor vesicles contained lipids labeled with two fluorophores (NBD and rhodamine) at quenching concentrations; the fusion with unlabeled acceptor vesicles resulted in dilution and dequenching of NBD. Drosophila ATL resulted in efficient fusion in the presence of GTP and Mg^2+^ (Fig. 5B) as reported previously (Bian et al., 2011). Consistently, no fusion was seen with GDP, GppNp, or the absence of Mg^2+^ (Fig. 5B). Surprisingly, human ATL1 was inactive under the same conditions, even with higher protein concentrations in the proteoliposomes compared to Drosophila ATL (Fig. 5B).

Because the human and Drosophila cytATLs exhibit equivalent GTPase activity, the TM and CT of Drosophila may convey more efficient membrane fusion *in vitro*. To test this hypothesis, we replaced the TM-CT of human ATL1 with that of Drosophila ATL and measured the GTPase activity and membrane fusion of the chimera. As expected, the detergent-solubilized and purified ATL chimera hydrolyzed GTP at the same rates as Drosophila and human ATL (Fig. 5C). However, membrane fusion was still not detected (Fig. 5B). These results suggest that fusion mediated by ATLs requires coordination between different domains of the molecule.

## Discussion

Drosophila ATL has played important roles in understanding homotypic ER fusion mediated by the ATL family, not only because fruit flies serve as a great genetic model for probing the physiological role of ER fusion, but also because it enables a powerful system for studying homotypic membrane fusion *in vitro*. Our results here provide new insights into ATL-mediated fusion and clarify a few possibilities for further understanding this process.

The crystal structure of Drosophila ATL in complex with GDP forms an unexpected second dimer form similar to human ATL1 in the “pre-fusion” state. This observation does not necessarily mean that ATL will switch from “post-fusion” to “pre-fusion” directly in the presence of GDP, but rather raises the possibility that GDP-bound ATL is simply flexible and may adopt multiple conformations before releasing GDP and recycling GTPase. Previous results indicated that ATL remains a relatively loose dimer in the presence of GDP (Bian et al., 2011). Our new data with both human and Drosophila cytATLs tested at a lower concentration clearly showed that GDP-bound ATL is mostly monomeric. These results favor the notion that the transition from dimer to monomer after GTP hydrolysis will occur spontaneously.

Drosophila ATL and human ATL1 have very high sequence similarity (Bian et al., 2011). Almost all key residues identified so far in the ATL family can be found in both ATLs. Thus, human ATL1 is expected to exhibit fusion activity *in vitro*, just like Drosophila ATL. Surprisingly, our results reveal that human ATL1 is inactive, even under the same conditions as Drosophila ATL. Structural and biochemical analyses revealed that GTPase activity is critical for the fusion reaction. In addition, the TM and CT regions of the molecule play important roles. However, none of these factors seem to be the determinants of the *in vitro* fusogenic activity of human ATL1. First, human ATL1 has same rate of GTP hydrolysis as Drosophila ATL, which is active in *in vitro* fusion. Second, the TM-CT of Drosophila ATL could not help human ATL1 with its fusion reaction when the active N-terminal cytosolic domain of human ATL1 is attached. Based on the depletion phenotype and the results of the yeast *in vivo* fusion assay (Hu et al., 2009; Anwar et al., 2012), human ATL1 is clearly an active ER fusogen *in vivo*. Unfortunately, it appears that the ATL activity in the *in vitro* fusion assays cannot be predicted based on its *in vivo* behavior.

The lack of *in vitro* fusion activity for human ATL1 suggests that it may be a less active fusion enzyme than Drosophila ATL, but its activity is elevated by unclear mechanisms *in vivo*. These two ATLs behave differently in cells in at least two respects. First, depletion of Drosophila ATL causes ER fragmentation (Orso et al., 2009), but depletion of human ATL does not. Second, Drosophila ATL tends to form puncta around the junctions of the tubular ER network (Moss et al., 2011), but human ATL1 is evenly distributed along ER membranes. Whether these differences are linked to the different *in vitro* activities of the two ATLs remains to be investigated.

## MATERIALS AND METHODS

### Protein expression, purification, and crystallization

The cytosolic domain of human ATL-1 (residues 18–447) and full-length Drosophila ATL were expressed and purified as described previously (Bian et al., 2011). The cytosolic domain of Drosophila ATL (cytATL; residues 1–413) was cloned into the pET-28a vector, expressed, and purified in the same manner as human ATL1. The full-length human ATL1 and chimeric ATL were amplified and cloned into the pGEX-6P-1 vector and expressed in *Escherichia coli* as GST fusion proteins. The proteins were purified in the same manner as full-length Drosophila ATL, and the purified proteins were concentrated to 1 mg/mL for subsequent analysis.

Drosophila cytATL protein (6 mg/mL) was incubated with 1 mmol/L GDP for 1 h at 4°C, and then crystallized using the hanging drop vapor diffusion method equilibrated against a reservoir solution of 22% PEG3350, 0.1 mol/L Tris, 0.2 mol/L NaCl, pH 8.5. Crystals were grown for one week at 20°C.

### Data collection and structure determination

Diffraction data for the crystal were collected at 100 K using a wavelength of 1.54 Å at a home-source X-ray facility (Rigaku MicroMax007HF) at Nankai University. For cryo-protection, the crystals were soaked in mother liquor plus 20% (*v*/*v*) glycerol for a few seconds before data collection. All data sets were processed with HKL2000 and converted to CCP4 format. The structure was determined by molecular replacement using the human ATL1 structure (Protein Data Bank code 3QOF) as a search model. The preliminary models were built and refined with the Phenix program.

### GTPase activity assay

GTPase activity was determined using the EnzChek phosphate assay kit (Invitrogen). Reactions were performed in a 100 µL volume and initiated by the addition of 0.5 mmol/L GTP. Absorbance at 360 nm was measured at 1-min intervals for 30 min at 37°C.

### Analytical ultracentrifugation

Sedimentation velocity data were collected at a speed of 142,249 ×*g* in an An-60 Ti rotor at 4°C. The proteins were prepared in 50 mmol/L Tris (pH 8.0), 5 mmol/L MgCl_2_, 1 mmol/L DTT, and 1 mmol/L nucleotide. Data were analyzed by the program SEDFIT (Version 11.8).

### Flotation assay

The reconstitution efficiency was determined by flotation of proteoliposomes on a sucrose gradient. Proteoliposomes (30 µL) were mixed with 100 µL of 1.9 mol/L sucrose and overlaid with 100 µL of 1.25 mmol/L sucrose and 20 µL of 0.25 mmol/L sucrose, all in 25 mmol/L HEPES (pH 7.5). The samples were centrifuged in a Beckman TLS 55 rotor at 55,000 rpm and 4°C for 75 min. The gradient was fractionated into five 50-µL fractions and analyzed by SDS-PAGE stained with Coomassie.

### Fusion assay

The *in vitro* lipid-mixing assay was performed as described previously (Bian et al., 2011). In brief, the full-length ATL proteins were reconstituted into the liposome, and the labeled donor proteoliposomes were mixed with unlabeled acceptor proteoliposomes in the presence of 5 mmol/L Mg^2+^ in a total volume of 100 µL per reaction. The fusion reaction was started by the addition of 0.5 mmol/L GTP and NBD fluorescence measured at 1-min intervals at 37°C. After 30 min, 5 µL of 10% (*w*/*v*) DDM was added to determine the total NBD fluorescence. Fusion was expressed as the percentage of total fluorescence.

## ACCESSION CODE

Coordinates and structure factors have been deposited with RCSB accession code: 3X1D.
